# Maternal feeding practices, child eating behaviour and body mass index in preschool-aged children: a prospective analysis

**DOI:** 10.1186/1479-5868-7-55

**Published:** 2010-06-28

**Authors:** Jane E Gregory, Susan J Paxton, Anna M Brozovic

**Affiliations:** 1 School of Psychological Science, La Trobe University, Bundoora, Melbourne, Victoria 3086, Australia

## Abstract

**Background:**

Previous research has found associations between parental feeding practices and children's eating behaviour and weight status. Prospective research is needed to elucidate these relationships.

**Methods:**

One hundred and fifty-six mothers of 2- to 4-year-old children completed questionnaires including measures of maternal feeding practices (pressure to eat, restriction, monitoring and modelling of healthy eating), child eating behaviour (food responsiveness, food fussiness and interest in food), and mother reported child height and weight. The questionnaire was repeated 12 months later. Regression analyses were used to find longitudinal associations between maternal feeding practices, child eating behaviour and child body mass index (BMI).

**Results:**

Modelling of healthy eating predicted lower child food fussiness and higher interest in food one year later, and pressure to eat predicted lower child interest in food. Restriction did not predict changes in child eating behaviour. Maternal feeding practices did not prospectively predict child food responsiveness or child BMI.

**Conclusion:**

Maternal feeding practices appear to influence young children's eating behaviour but not weight status in the short term.

## Background

The World Health Organization has acknowledged a global "obesity epidemic", attributed to recent increases in the availability of energy-dense foods and sedentary lifestyles [1]. In line with international trends, the proportion of Australian children classified as overweight or obese rose from 11-12% in 1985 to 23% in 2007 [2,3]. Despite global environmental changes, there are still large variations in weight status between children, indicating that there are individual differences in children's susceptibility to the current obesogenic environment [4]. Differences may be physiological (e.g., metabolism), behavioural (e.g. responsiveness to food) or environmental (e.g., parental feeding practices). It is essential to explore modifiable factors that may contribute to child weight status to inform the development of effective public health interventions.

Parental feeding practices refer to the behaviours of parents that influence children's eating. Strategies used by parents might include direct attempts to control children's food intake, such as pressuring children to eat more, or restricting their intake of unhealthy foods. Control may also be exerted indirectly by monitoring the child's intake of unhealthy foods, or by modelling healthy eating in front of the child [5,6]. The effectiveness of these strategies as a means to modify child eating behaviour or child weight status is unclear.

Cross-sectional studies have found that both pressure to eat and restriction were associated with child eating behaviour and child weight status. Pressure to eat has been consistently associated with lower child body mass index (BMI) [7-10] and has been found to be more frequently used by parents whose children are fussier eaters [11,12]. In turn, children's fussy eating and rejection of new foods have been associated with lower BMI [13,14], less healthy food preferences and poorer nutrition [15,16]. Parental use of restriction has been associated with higher child weight in some studies [10,17], but not others [8,18]. Restriction was also related to girls' eating in the absence of hunger [19], an eating style that is positively associated with BMI [13,14]. Monitoring, on the other hand, has not been associated with child BMI [8,10] or eating behaviours [11] in cross-sectional studies. Due to the cross-sectional nature of these studies, we cannot draw conclusions about possible causal relationships. Parents' use of pressure, restriction and monitoring may be reactive to the eating behaviour or BMI of their children, or alternatively these feeding practices could contribute to the development or maintenance of eating behaviour and weight status.

A small number of studies have examined the longitudinal impact of parental feeding practices on child eating behaviour and weight. Faith et al. [20] conducted a prospective analysis of parental feeding practices in a sample of 57 parents of 7-year-old children. Children were categorised as either high or low risk for overweight according to whether their mothers' pre-pregnancy weight was above the 66^th ^or below the 33^rd ^percentile. The authors found that higher child BMI at 7 years was predicted by lower use of pressure to eat and higher use of restriction by parents at 5 years, but only in children who were at a high risk for overweight. In the low risk group, more frequent use of monitoring at 5 years predicted lower BMI when the child was 7 years old. These relationships remained significant after controlling for the child's BMI at 3 years [20]. In contrast, a study of 74 white children and 47 African American children revealed no longitudinal impact of pressure to eat, restriction or monitoring on changes in total fat mass over 2.7 years [21].

With regards to child eating behaviour, one study found that mothers who used pressure to eat more frequently when their daughters were aged 7 years, had daughters who were pickier eaters at age 9 years. This study also found mothers' fruit and vegetable consumption at 7 years was associated with higher fruit and vegetable consumption and lower picky eating in daughters at 9 years. The results suggested that modelling of healthy eating was a more effective strategy than pressure to promote healthful eating behaviour [15]. Another study with a sample of 9-year-old girls found that girls were more likely to eat in the absence of hunger at 9 years when their mothers used high levels of restriction at 5 years, compared with those whose mothers used low levels of restriction [22].

Together, these studies provide some evidence that pressure to eat and restriction may be counter-productive feeding strategies. It seems intuitive to use pressure to increase children's consumption of healthy foods, and restriction to reduce their consumption of unhealthy foods, but these feeding practices may actually induce the opposite of the intended effect. Further research using broader samples is required to explore these relationships further.

We were unable to find any other prospective studies addressing the impact of modelling of healthy eating on child eating behaviour or BMI. However, a number of studies have found that parents' fruit and vegetable consumption is positively related to child fruit and vegetable consumption [23-25]. Furthermore, experimental research has found that toddlers and pre-school aged children were more likely to accept a new food if an adult was eating the same food, than they were if the food was simply presented to them [26,27]. Further research is required to establish whether modelling of healthy eating has a sustained positive impact on child eating behaviour, and subsequently weight.

This brief review of relationships between parental feeding practices, child eating behaviour and child BMI has highlighted the need for more prospective research in this area. There is some evidence to suggest that pressure to eat and restriction may have a negative impact on child eating behaviour, which could affect child weight status over time. However, these preliminary conclusions are based on a small number of studies with primary school-aged children. A large twin study suggested that child eating behaviours are already established by the age of 4 years, and remain stable thereafter [28], and so young children are a particularly important target group for identifying the risk and protective factors for healthy eating.

The aim of the present study was to examine the extent to which maternal feeding practices prospectively predict the development of eating behaviour and BMI in pre-school-aged children. We hypothesised that the use of maternal feeding practices with 2- to 4-year-old children would prospectively predict child eating behaviours and child BMI over 12 months. We expected that these relationships would be significant after controlling for child age and gender, for maternal age, BMI and education, and for initial levels of the dependent variable (i.e., the particular child eating behaviour or child BMI at time 1).

Specifically, based on previous research we hypothesised that over a period of 12 months:

• Pressure to eat would be associated with higher child food fussiness and lower child interest in food.

• Modelling healthy eating would be associated with lower child food fussiness and higher child interest in food.

• Restriction would be associated with higher child food responsiveness.

• Monitoring would be associated with lower child food responsiveness.

• Pressure to eat would negatively and restriction would positively prospectively predict child BMI.

## Methods

### Participants and procedure

The sample consisted of mothers of children aged between 2 and 4 years old from The Child and Family Health Study in Melbourne, Australia [29]. Recruitment was conducted through community notices in local newspapers and via playgroup co-ordinators who invited mothers in their groups to participate. Mothers were instructed to complete the questions with regard to one specific child at both time points, and at follow up they were provided with the initials and date of birth of the child they had initially responded about. Questionnaires were received from 183 mothers in the initial phase, and 157 (86%) of these participated in the 12-month follow up. One participant was removed due to incomplete data on several subscales, leaving a final sample of 156.

This study was approved by the Human Research Ethics Committee at La Trobe University. Participants were sent two identical questionnaires one year apart. The mean period between Time 1 (T1) and Time 2 (T2) was 55 weeks (standard deviation = 4 weeks), with a range of 39 to 67 weeks. Participants received a $10 supermarket gift voucher for their participation at each time point.

### Measures

#### Demographic information and Body Mass Index

The demographic questionnaire included information about the mother's age and level of education, as well as height and weight information for calculating her BMI. Mothers also reported on their child's age, gender, weight and height. Child BMI *z *scores (BMIz) were calculated using NutStat, a computer program which calculates BMIz adjusted for age and gender, using United States based norms from the Center for Disease Control and Prevention (CDC) 2000 growth charts [30].

#### Feeding practices and eating behaviour

Three subscales from the widely used Child Feeding Questionnaire (CFQ) were used to measure maternal feeding practices: pressure to eat; restriction; and monitoring [5].

Modelling of healthy eating was measured using three items written for the purposes of this study: "I try to eat only healthy foods in front of my child"; "My child sees me eating fast food" (reversed item), and; "My child sees me eating healthy snacks (e.g. fruit, yoghurt, nuts, toast)." These were measured on a 5-point Likert scale from 1 = never, to 5 = always.

Child eating behaviours were measured using items from the food responsiveness and food fussiness subscales of the Child Eating Behaviour Questionnaire (CEBQ), which has been shown to have good validity and internal reliability [31,32]. The food responsiveness subscale measures the extent to which children eat in response to food cues rather than satiety (e.g. "Even if my child is full up s/he finds room to eat his/her favourite food"). Food fussiness measures children's picky eating behaviour (e.g. "My child is difficult to please with meals") and acceptance of new foods (e.g. "My child enjoys tasting new foods").

### Data analysis

To establish that the eating and feeding measures were appropriate for our sample, a factor analysis was conducted on each subscale. The original items from a subscale were retained when the scree plot showed one predominant factor with an eigenvalue greater than one. Cronbach's alpha (α) was used to test internal reliability of subscales. Variables were created by calculating the mean of all items in a subscale, and missing items were replaced with the participant's mean subscale score. Correlation analyses were used to measure stability of maternal feeding practices and child eating behaviours across the two time points.

Associations between maternal feeding practices, child eating behaviours and child BMIz were tested using Pearson's correlation coefficients and hierarchical multiple regressions. We first tested T1 and T2 cross-sectional correlations between feeding eating and BMIz, and then cross-lag correlations between T1 and T2. Hierarchical multiple regressions were used to test whether maternal feeding practices could predict changes in child eating behaviours over time. Time 2 child eating behaviours were entered as dependent variables, with a separate analysis for each of the three behaviours (food responsiveness, food fussiness, and interest in food). At step 1, we controlled for the respective T1 eating behaviour, and for potential covariates of maternal age, BMI and education, and child age and gender (1 = male; 2 = female). At step 2, we entered T1 pressure to eat, restriction, monitoring and modelling. If the T1 feeding practices were significant predictors of T2 eating behaviour after taking into account the effect of prior (T1) eating behaviour, this would indicate support for the hypothesis that maternal feeding practices predict the development of particular child eating behaviours.

A similar process was used to determine whether there was a longitudinal effect of maternal feeding or child eating on child BMI *z*-scores. T2 child BMIz was entered as the dependent variable. T1 child BMIz and the same potential co-variates were controlled for at step 1 before entering predictor variables at step 2. Two analyses were conducted, with maternal feeding practices (pressure to eat, restriction, monitoring and healthy modelling) as the independent variables in one analysis, and child eating behaviours (food responsiveness, food fussiness and interest in food) in the other.

## Results

### Participant characteristics

The final sample consisted of 156 mothers, who were aged between 22 and 48 years (M = 35, SD = 5.2) at the point of initial recruitment. Eighty-seven percent of mothers were married or in a de facto relationship, and 60% were tertiary educated. Of the 137 (88%) mothers who provided height and weight information, 51% could be categorised as overweight or obese (BMI > 25), which is consistent with other Australian data for this age group [33].

The children of the participants were 51% female and had a mean age of 3.3 years (SD = 0.8) at the point of initial recruitment. Complete height and weight information for both time points was available for 68% of the children. Of these children, 15% could be considered overweight or obese according to the cut-off points of the International Obesity Task Force [34]. This represents a lower proportion of overweight in our sample when compared with age-matched peers in the Australian population [3]. Independent samples *t *tests found that those who did and those who did not provide height and weight data for their children did not significantly differ in age, mother's education, BMI, or any of the eating and feeding subscales (data not shown). The full sample (N = 156) was therefore retained, with a subsample (n = 106) used for the analyses requiring child height and weight data.

### Scale properties and factor analysis

A separate principal components analysis (PCA) was conducted on each subscale to ensure that all items loaded onto one unique factor. For each of the CFQ pressure to eat and monitoring subscales, a single factor was found explaining 59% and 80% of the variance, respectively, and Cronbach's alphas (α) were .77 and .87. The restriction subscale PCA showed three factors with eigenvalues greater than one, but the screeplot showed a clear break after two factors, indicating that the first two factors explained much more of the variance than the remaining factors. The PCA was run again, this time forcing the items to load onto two factors. Two of the eight items did not load onto the first factor, so these two items (both regarding the use of food as a reward for good behaviour) were removed, and the final PCA showed one distinct factor explaining 50% of the variance. This 6-item version was used in the final analyses (α = .80). The three items measuring modelling of healthy eating all loaded onto one unique factor with an eigenvalue greater than one, explaining 63% of the variance. Unrotated items loadings ranged from .73 to .85 (α = .70).

The five items from the CEBQ food responsiveness subscale loaded onto a unique factor, explaining 54% of the variance (α = .76), and so all items were retained. A PCA of the six food fussiness items showed two discrete variables, explaining 41% and 36% of the variance. A varimax rotatation revealed that there were three items in each factor, and examination of the items found a distinct difference in language between the two factors. One referred to the child refusing new foods and being difficult to please with meals, while the other used positive language regarding the child's openness to new foods and enjoyment of variety in food. For the final analyses we kept these factors separate, labelling them food fussiness (α = .81) and interest in food (α = .89), respectively.

Mean scale scores for both time points are presented in Table 1. Normality was assessed using skewness and kurtosis statistics as well as visual inspection of histograms. All feeding and eating measures were found to be acceptable for use in regression analysis, with the exception of the monitoring subscale, which was corrected by using a logarithmic transformation for this variable in all correlation and regression analyses.

**Table 1:** Mean (s.d.) scores and bivariate correlations for time 1 and time 2 maternal feeding practices and child eating behaviour (*N *= 156)

	Mean (s.d.)		Pearson's *r*
		
	Time 1	Time 2	
	(Mean age 3.3 years)	(Mean age 4.3 years)	
Pressure to eat^a^	2.74 (1.10)	2.73 (1.08)	.69 (*p *< .001)
Restriction^a^	3.55 (0.94)	3.35 (1.08)	.59 (*p *< .001)
Monitoring^a^	4.45 (0.71)	4.33 (0.74)	.53 (*p *< .001)
Model healthy	3.94 (0.58)	3.87 (0.57)	.73 (*p *< .001)
Food responsiveness^b^	2.43 (0.70)	2.49 (0.70)	.62 (*p *< .001)
Food fussiness^b^	2.96 (0.84)	3.11 (0.81)	.64 (*p *< .001)
Interest in food^b^	3.33 (0.87)	3.19 (0.81)	.21 (*p *< .05)

^a ^Child Feeding Questionnaire

^b ^Child Eating Behaviour Questionnaire

### Stability of feeding and eating behaviour

Correlations between the two time points were highly significant (p < .001) for all of the feeding and eating subscales, with the exception of interest in food, which had a lower but still significant correlation (*r *= .21, *p *< .05). These results are presented in Table 1.

### Simple associations between maternal feeding, child eating and child BMI

Cross-sectional correlation analyses between maternal feeding, child eating and child BMI were conducted for both time points (Table 2). More frequent use of pressure to eat was associated with higher child food fussiness and lower child interest in food at both time points. Restriction was linked with higher food responsiveness at both time points, and with higher fussiness and lower interest in food at T2. Modelling of healthy eating was positively correlated with interest in food at T2, but not T1. At T1, higher child BMIz was associated with more food responsiveness, and less frequent maternal pressure to eat.

**Table 2:** Cross sectional correlations between maternal feeding, child eating and child BMI at time points 1 and 2 (*N *= 156)

	Child eating behaviours							
	Time 1 (Mean age 3.3 years)				Time 2 (Mean age 4.3 years)			
		
	**FR**^**b**^	**FF**^**b**^	**IF**^**b**^	BMIz	**FR**^**b**^	**FF**^**b**^	**IF**^**b**^	BMIz
				(*n *= 106)				(*n *= 106)
Pressure to eat^a^	-.09	.25**	-.21**	-.20*	-.06	.30**	-.41**	-.02
Restriction^a^	.34**	.12	-.13	.09	.27**	.25**	-.18*	.07
Monitoring^a^	-.02	-.06	.04	.08	-.02	-.12	.15	-.06
Model healthy	-.14	-.11	.14	-.05	-.12	-.14	.22**	-.01
BMI *z*-score	.20*	-.14	.00		.19	.08	-.02	

Abbreviations: FR, food responsiveness; FF, food fussiness; IF, interest in food; BMIz, body mass index *z*-score

*p < .05 **p < .01

^a ^Child Feeding Questionnaire

^b ^Child Eating Behaviour Questionnaire

### Cross-lag associations between maternal feeding, child eating and child BMI

Correlation analyses were used to measure whether T1 maternal feeding behaviours were associated with T2 child eating behaviour and BMIz (Table 3). Pressure to eat at T1 was associated with higher food fussiness and lower interest in food at T2. Restriction at T1 was positively correlated with food responsiveness at T2. More frequent use of modelling of healthy eating at T1 was associated with less food responsiveness and fussiness, and more interest in food at T2.

**Table 3:** Cross-lag correlations between maternal feeding, child eating and child BMI (*N *= 156)

	Time 2 Child eating behaviours			
	(Mean age 4.3 years)			
Time 1	**FR**^**b**^	**FF**^**b**^	**IF**^**b**^	BMIz
(Mean age 3.3 years)				(*n *= 106)
Pressure to eat^a^	-.03	.22**	-.32**	.03
Restriction^a^	.24**	.13	-.10	.10
Monitoring^a^	-.11	-.05	.08	-.01
Model healthy	-.16*	-.27**	.29**	-.05

Abbreviations: FR, food responsiveness; FF, food fussiness; IF, interest in food; BMIz, body mass index *z*-score

*p < .05 **p < .01

^a ^Child Feeding Questionnaire

^b ^Child Eating Behaviour Questionnaire

### Longitudinal analysis

Hierarchical multiple regressions were used to test whether maternal feeding practices could predict changes in child eating behaviours over time (Table 4). The combined model of prior eating behaviour and potential covariates (child age and gender, and maternal age, BMI and education) (step 1) was a significant predictor of both food responsiveness and food fussiness, explaining 42% and 46% of the variance for the two eating behaviours, respectively. Prior child eating behaviour was the only significant independent predictor at step 1 for both food responsiveness and fussiness. Maternal feeding practices significantly added to the model for food fussiness, but not food responsiveness (step 2). Modelling of healthy eating at T1 negatively predicted child food fussiness at T2.

**Table 4:** Longitudinal predictors of child eating behaviours (*N *= 156)

	Time 2 Eating behaviour (Mean age 4.3 years)		
	
Time 1 predictors		Standardised Beta (*p*)	
	
(Mean age 3.3 years)	**Food responsiveness**^**b**^	**Food fussiness**^**b**^	**Interest in food**^**b**^
*Step 1*	*R*^*2*^_*Change *_*= .42 (p < .001)*	*R*^*2*^_*Change *_*= .46 (p < .001)*	*R*^*2*^_*Change *_*= .06 (n.s.)*
Eating behaviour^c^	**-.63 *(< .001)***	**.67 *(< .001)***	**.22 *(.014)***
Child age	.12 *(n.s.)*	-.14 *(n.s.)*	.03 *(n.s.)*
Child gender	-.12 *(n.s.)*	-.10 *(n.s.)*	.10 *(n.s.)*
Maternal age	-.04 *(n.s.)*	-.09 *(n.s.)*	.01 *(n.s.)*
Maternal BMI	.07 *(n.s.)*	.02 *(n.s.)*	-.07 *(n.s.)*
Maternal education	.02 *(n.s.)*	.06 *(n.s.)*	-.03 *(n.s.)*
*Step 2*	*R*^*2*^_*Change *_*= .01 (n.s.)*	*R*^*2*^_*Change *_*= .04 (p < .05)*	*R*^*2*^_*Change *_*= .13 (p < .05)*
Pressure to eat^a^	.00 *(n.s.)*	.01 *(n.s.)*	**-.25 *(.005)***
Restriction^a^	.06 *(n.s.)*	.04 *(n.s.)*	-.02 *(n.s.)*
Monitoring^a^	-.09 *(n.s.)*	.07 *(n.s.)*	-.04 *(n.s.)*
Model healthy	-.01 *(n.s.)*	**-.23 *(.002)***	**.25 *(.010)***

Abbreviation: n.s., not significant (p > .05)

^a ^Child Feeding Questionnaire

^b ^Child Eating Behaviour Questionnaire

^c ^Refers to the Time 1 measurement of the same eating behaviour variable used as the Time 2 outcome variable

Child interest in food at T2 was not significantly predicted by the model combining potential covariates and prior child interest in food (step 1). The combined maternal feeding practices variables entered at step 2 significantly added to prediction of the model, explaining 13% of the variance. Pressure to eat at T1 negatively predicted child interest in food at T2. Modelling of healthy eating at T1 positively predicted interest in food at T2.

For child BMIz, the model combining potential maternal and child covariates with T1 BMIz (step 1) was a significant predictor of T2 BMIz, explaining 25% of the variance *(R*^*2 *^*= .25, p < .001)*. Child's BMIz at T1 was the only significant independent predictor (*β *= .49, *p *< .001). BMI *z*-score at T2 was not significantly predicted by T1 maternal feeding practices (*R*^*2*^_*Change *_*= .01, p = .857)*, or by T1 child eating behaviours (*R*^*2*^_*Change *_*= .01, p = .707*) after controlling for maternal and child covariates, and T1 BMIz.

## Discussion

The aim of this study was to examine whether maternal feeding practices predicted child eating behaviours over one year after controlling for initial child eating behaviours in pre-school children. Our first hypothesis was partially supported. We found that pressure to eat negatively predicted interest in food but there was no prospective relationship between pressure to eat and food fussiness. Our second hypothesis, that modelling healthy eating would prospectively negatively predict food fussiness and positively predict interest in food was supported. Our third and fourth hypotheses that restriction would positively and monitoring would negatively predict food responsiveness was not supported. The secondary aim was to establish whether maternal feeding practices or child eating behaviours would predict child BMIz one year later, after controlling for initial BMIz. Contrary to our fifth hypothesis, there were not significant prospective predictors of child BMIz.

The finding that pressure to eat did not predict decreased child food fussiness 12-month period was contrary to both the original hypothesis, and the results of a prior longitudinal study, which found that girls whose mothers used higher levels of pressure to eat when they were 7 years old, showed more fussy eating behaviour at 9 years old [15]. There are a number of discrepancies between that study and the present research that could account for the different finding. The difference in ages between the two samples or the shorter time lapse between the two points of measurement may have influenced the findings. Unlike the present study, Galloway et al. [15] did not control for levels of fussy eating at the first time point, so it is not clear whether the pressuring techniques actually caused a change in girls' eating behaviour over time. The positive association between pressure at age 7 and fussy eating at age 9 might be explained by the child's initial fussy eating tendency, to which the parents responded with pressure to eat. Another reason for the distinction may be that the present study distinguished between fussy eating and interest in food. Pressure to eat at Time 1 was associated with the child's decreased interest in new foods over the 12-month period.

Batsell and colleagues [35] suggested that children who are forced to consume certain foods develop a "cognitive aversion" for those foods because they associate the food with the negative feeding experience. It is possible that the cognitive aversion might also apply to the general experience of trying new foods. If trying a new food is consistently paired with the occurrence of being forced to eat an unpalatable food, it may be that a child would show less interest in trying new foods in the future.

Previous research has found that parental consumption of fruits and vegetables is associated with child consumption of these foods [23-25]. This has been attributed to social facilitation, a process in which children are more likely to engage in a behaviour if others present are engaging in the same behaviour [36]. This is supported by experimental research finding that children are more likely to taste a new food if other people are eating the same food than if the food is simply presented to them [26,27,37]. In the present study, we found that modelling of healthy eating was associated with decreased food fussiness and increased interest in foods over the 12-month period. It is possible that the child's experience of trying of new foods in the modelling situation is positively reinforced by the pleasant physical feelings experienced when they eat the healthy foods that are being modelled for them, making them more likely to try new foods again in the future. Another possibility is that the behaviour is reinforced through a cognitive pairing, similar to the "cognitive aversions" proposed by Batsell et al. [35]. If children are enjoying the shared experience of eating together with their parents in the modelling situation, then it is possible that these positive emotions could serve as a reinforcer for the trying of new foods.

The items for the two measures, food fussiness and interest in food, originated from the single measure of food fussiness from the Child Eating Behaviour Questionnaire. A principle components analysis showed that the items clearly loaded onto two factors. For our sample, there was a distinct difference in the reporting of the negative behaviour of refusing foods, and the positive behaviour of enjoying trying new foods. This may have been a reflection of the language used in the measurement tool, or it could be an indication that an active enjoyment in trying new foods is not necessarily the opposite end of the fussiness continuum. An interest in trying new foods is a beneficial trait for a child to have, because it can take repeated tastings of a new food before a child accepts it [38]. It is likely that children who are interested in trying new foods will accept and enjoy a broader range of foods and will be more likely to meet their nutritional needs, and less likely to resort to more palatable, high-energy foods.

We did not find a significant longitudinal relationship between maternal use of restriction and child food responsiveness. This was contrary to both our hypothesis and prior research by Birch, Fisher & Davison [22], who found that mothers using more frequent restriction when their daughters were aged 5 had girls with higher levels of eating in the absence of hunger at aged 9. Again, there are a number of distinctions between the two studies that could explain the difference in the findings. It could be that younger children are more resilient to the effects of restriction. There is some evidence to suggest that younger children are able to regulate the eating according to internal hunger and energy intake than their older counterparts [28]. While we controlled for child age in all regressions in the present study, doing this would not take into account the differences in eating behaviour in our preschool-aged sample, compared with the 9-year-olds in Birch et al.'s study. We also used a different measure for eating behaviour, making it difficult to compare the two studies. Furthermore, our study only explored the influence of feeding practices after one year, and it could be that it takes longer than that to see effects on child eating behaviour.

In the present study, cross-sectional analyses revealed that restriction was positively correlated with food responsiveness at both time points. This finding, combined with the lack of a significant prospective association, provides support for the theory that restriction is a technique used in response to a food responsive eating style, and not necessarily a cause of it. However, it is important to be aware that these correlations could also be explained by third factor common to both variables, such as maternal concern about child weight status. Some recent studies have found an association between maternal use of restriction and concern about weight, but not between restriction and actual child weight status [18,39]. Considering that parents tend not to be able to accurately recognise when their child is overweight [39], it could be that a combination of the child's actual weight and the child's food responsive eating style elicits concern in the parent, who then uses restrictive feeding practices in an attempt to modify the child's eating behaviour and subsequently weight. It is important to note that while we did not find any increase in food responsiveness or child weight, we also did not find any decrease, indicating that for this sample, restriction was not an effective modification tool.

We did not find any maternal feeding practices or child eating behaviours that predicted child BMI over time. It was interesting to note that in our sample, only 25% of the variance in child BMI at time 2 could be explained by the combined model of T1 child BMI, maternal BMI, age and education, and child age and gender. Of these, prior child BMI was the only independent predictor. A recent Australian study also found no significant association between maternal BMI and child BMI, and suggested that this was due to the young sample [40]. These findings indicate that environmental factors play a key role on child weight in these early years, highlighting the importance of understanding early influences on both child weight and on the eating habits that may later contribute to weight status.

This study benefitted from a longitudinal design, with measures repeated at each time point so that we were able to examine cross-sectional data at both time points in additional to the prospective analysis. We examined a range of feeding practices, including modelling of healthy eating which had not previously been studied in a prospective study with pressure, restriction and monitoring. Our 2- to 4-year-old sample enabled us to capture a key developmental period for child eating behaviour. There were, however, some limitations to the design. We had a relatively small sample for the number of comparisons we made, which increases the likelihood of finding a false significant result [41]. More than half our sample was tertiary educated, indicating that we may not have adequately captured a representative sample of women from a range of socio-economic backgrounds. Some studies show higher rates of overweight and obesity in people from a lower socioeconomic status [42], suggesting that these groups should be targeted for research and interventions. Our results should be generalised with caution, although it should be noted that the rates of overweight in our sample were in line with estimated national averages. Another limitation with our study was that we relied on mothers' reports rather than direct observations. Some mothers responses may have been influenced by their perceptions of the "right" behaviour with regards to feeding their children, and social desirability with regards to maternal and child weight status. There is evidence to suggest that parents tend to under report the weight of overweight children [43], which could explain the lower proportion of overweight and obese children (15%) in our sample compared with data for age-matched peers in Australia (18-21% for 2-3 year olds in 2007) [3]. Finally, our measure of modelling of healthy eating was one written for the purposes of this study. Items were written from a theoretical basis and relied on mothers' perceptions of what constitutes "healthy foods". The measure has not been independently validated, although is did have sufficient internal consistency and showed associations in accordance with our predictions. Given the significant findings for this variable, it would be useful to explore the concept of healthy modelling further, and to test whether this is a valid and reliable measure for modelling of healthy eating.

Despite the recent increases in childhood overweight and the current obesogenic environment [1,44], the majority of children in Australia are able to maintain a healthy weight and meet their nutrient requirements [3]. The rate of overweight in adults, on the other hand, is more than twice that of children [33]. While there is evidence to suggest that eating behaviours developed in childhood carry on into early adulthood [45], perhaps the weight and health consequences of these behaviours don't become evident until later in childhood, as parental capacity as gatekeeper over the child's diet is reduced.

## Conclusions

Our results suggest that modelling of healthy eating towards children has a positive effect over time on young children's acceptance of new foods and interest in a variety foods, however further research is required to establish the most appropriate time and means for preserving a child's natural ability to respond to internal cues of satiety and to adjust their eating according to energy intake. Parents may benefit from public health interventions that promote adult healthy eating habits as a means for developing and maintaining healthy eating behaviour in their children.

## Competing interests

The authors declare that they have no competing interests.

## Authors' contributions

Original construct devised by JEG. JEG, SJP and AMB contributed to study design and questionnaire compilation. JEG and AMB recruited participants and conducted data collection. JEG completed data analysis and drafted the manuscript. JEG and SJP revised subsequent versions of the manuscript. SJP supervised The Child and Family Health Study. All authors read and approved the manuscript.
